# Duncan Wood

**Published:** 1970-07

**Authors:** 


### Duncan WOOD, M.B., B.S., F.R.C.S.

Mr. Duncan Wood, formerly Consultant Surgeon to the Royal Hospital, Bristol, Cossham Memorial Hospital, Bristol, and to Bridgwater Hospital, Somerset, died on July 24th, 1969, at the age of 82.

He was born on September 2nd, 1886, at Corsham, Wiltshire, where his father was in General Practice. He was educated at the Imperial Service College, which was at Windsor in those days. From there he went to the London Hospital and qualified in 1909. Three years later he became a Fellow of the Royal College of Surgeons, and after holding some house appointments, became Surgical Registrar and Senior Resident Officer at the Bristol General Hospital.

During the First World War he served as a regimental medical officer, and in a CCS, and then as a surgical specialist at a British Army Hospital. When he returned to Bristol, he met Eileen Holman, who was a nurse, and whom he married in 1922, shortly after his appointment to the staff of the Bristol General Hospital as Assistant Surgeon.

Their marriage was a very happy one, blessed with four sons, three of whom are now in General Practice, and the eldest son is a well known business man in Somerset.

To his profession as a General Surgeon, he devoted a long and hard working life. He lived before the age of special departments, so that he acquired a volume of experience and skill which is seldom found today. He was a sound and reliable teacher, popular with his students, who referred to him affectionately as 'Woody'. It was regarded as a great privilege to be his dresser, and better still his house surgeon.

He was much sought after as a consultant, and many practitioners in the West Country had cause to be grateful on numerous occasions, for his sound advice and practical skill. He also made useful contributions to medical literature.

A great sense of humour made him a popular colleague, and it was seldom if ever that he was put out about anything. A wonderfully balanced outlook on life enabled him to see something amusing or advantageous in many difficult situations.

In the Second World War he worked all through the air raids in Bristol as well as doing his ordinary work. A bomb fell one night in the road outside his house, blowing out all the windows, and causing a lot of superficial damage; he was very philosophical about it, and a few weeks later when one fell in his garden, not more than 25 feet or so from his house, he and the family were busy early next morning filling in the crater! To a colleague who called to see how things were with the family, his only comment was:— 'It's this levelling up process you see! Last time they blew the road into the garden, so this time they have blown the garden into the road!'

He was a man with many friends, and such charity of spirit that it is doubtful if anyone ever heard him say an unkind or uncharitable word—no matter how great the provocation.

After his retirement about the time the N.H.S. was born, he and his wife moved to Plymouth, and he took charge of the South West Regional Cancer Records Bureau. In 1953 he retired completely, and they moved to Hallatrow, just south of Bristol where he lived in happy retirement and looked after his beautiful garden. He was a tireless and very good gardener, and could be found out of doors doing something useful in almost all weather.

He played golf all his life except during the last few years, and many of his colleagues can recall happy times playing in his company. Although retired for many years before his death he was a regular attender at surgical and clinical meetings, and he always kept in touch with his numerous friends.

He was a member of the British Medical Association and also the Bristol Medico-Chirurgical Society.
G.M.F.

